# Domain-Specific Adult Sedentary Behaviour Questionnaire (ASBQ) and the GPAQ Single-Item Question: A Reliability and Validity Study in an Asian Population

**DOI:** 10.3390/ijerph15040739

**Published:** 2018-04-12

**Authors:** Anne H. Y. Chu, Sheryl H. X. Ng, David Koh, Falk Müller-Riemenschneider

**Affiliations:** 1 Saw Swee Hock School of Public Health, National University of Singapore, Singapore 117549, Singapore; ephsnhx@nus.edu.sg (S.H.X.N.); david.koh@ubd.edu.bn (D.K.); ephmf@nus.edu.sg (F.M.-R.); 2 PAPRSB Institute of Health Sciences, Universiti Brunei Darussalam, Jalan Tungku Link, Gadong BE1410, Brunei Darussalam; 3 Institute of Social Medicine, Epidemiology and Health Economics, Charité University Medical Centre Berlin, Berlin 10098, Germany

**Keywords:** accelerometers, measurement, psychometrics, questionnaires, validation

## Abstract

This study examined the validity and reliability of a domain-specific Adult Sedentary Behaviour Questionnaire (ASBQ) and the Global Physical Activity Questionnaire (GPAQ) single-item sitting question using self- and interviewer-administered modes of administration against the triaxial ActiGraph wGT3X-BT accelerometer. The ASBQ and the GPAQ were administered twice, seven days apart. Participants were asked to put on the waist-worn accelerometer for seven days. Convergent validity was assessed using Spearman’s rho, mean absolute error (MAE), and Bland-Altman analysis (*n* = 78). Reliability was assessed using the Spearman’s rho and intraclass correlation coefficient (ICC) (*n* = 84). Participants were adults aged 20–65 years and identifying as Chinese, Malay, or Indian. Only the self-administered GPAQ was significantly correlated with accelerometry-based measures (rho: 0.46), but not the interviewer-administered version (rho: 0.12). MAE for GPAQ was 207.5–218.3 min/day in relation to the accelerometer and for ASBQ was 154.7–174.6 min/day. Bland-Altman plots demonstrated large limits of agreement between questionnaire and accelerometry-based measures. While the self-administered GPAQ demonstrated a moderate correlation with accelerometry, the mean bias and the limits of agreement were large. The GPAQ (rho: 0.68–0.79; ICC: 0.68–0.78) and the ASBQ (rho: 0.53–0.64; ICC: 0.66–0.74) showed moderate-to-good reliability for total sedentary time using either self- or interviewer-administration. Future research should incorporate accelerometers to generate useful sedentary behaviour measures.

## 1. Introduction

Sedentary behaviour is defined as any waking behaviour that is characterized by an energy expenditure of ≤1.5 metabolic equivalent of tasks (METs), while in a sitting, reclining, or lying posture (sitting while using computers, watching television, and commuting using automobile at work or during leisure time) [1]. There is evidence from large cohort studies showing that sedentary behaviour *per se* is a distinct construct beyond lack of physical activity [2]. Such behaviours among modern humans have been associated with myriads of chronic metabolic disorders, excess adiposity and increased risks of mortality [3]. Individuals who meet the current physical activity guidelines, but at the same time are being highly sedentary for the rest of the day are termed “active couch potatoes”. It was found that even among individuals with high levels of physical activity, high levels of sedentary behaviour were still associated with detrimental metabolic risk factors [4] and mortality risks [5].

As prolonged sedentary time has become the default setting for many people [6,7], accurate measurements of sedentary behaviour are required in order to help develop interventional research and public health guidelines. Sedentary behaviour can be measured using subjective methods (e.g., questionnaire, recall instruments, etc.) and objective measures (e.g., accelerometry, inclinometer sensors, heart rate monitors, etc.) [8]. Accelerometry-based measures provide a convenient way to estimate objective data that are not subject to response and recall bias [9,10]. However, accelerometry-based measures are relatively costlier than questionnaires for data collectors and labor-intensive in large population-based studies [11]. Another challenge with accelerometry is that information about the context (domain) of sedentary behaviour is not easily captured by accelerometers, which is often done by questionnaires [10,12]. Questionnaires, on the other hand, are most widely used owing to their relatively low cost, minimal burden on participants, and higher applicability of use, especially in large-scale epidemiological studies [13].

A number of questionnaires to measure the various aspects of sedentary behaviour have been developed. In particular, the Global Physical Activity Questionnaire (GPAQ) consists of a single-item sitting question and has been extensively used in international population-based studies and national surveillance [14,15,16]. The GPAQ has been validated against accelerometry in Europe and other countries, such as South Africa, China (Shanghai), and Vietnam, with Spearman’s rho coefficients ranging from −0.02 to 0.40 for total sitting time [17,18,19]. The Adult Sedentary Behaviour Questionnaire (ASBQ) presented in the current study is a newly established instrument that measures total and context-specific sedentary behaviour. It was developed based on the previously validated Sedentary Behaviour Questionnaire (SBQ) [20], Sitting Questionnaire (SIT-Q) [21], last seven-day sedentary behaviour questionnaire (SIT-Q-7d) [22], and Workforce Sitting Questionnaire (WSQ) [23]. The ASBQ consists of seven items that are covering aspects of sedentary behaviour across the work-, transportation, and leisure-time domains. Collecting more composite information of sedentary time is useful for the development of targeted interventions [24,25,26]. As questionnaires can be subject to bias that is attributable to perceived cultural norms, social desirability, or recall bias [8], there is a need to develop reliable and valid questionnaires to be used in the Asian context.

Furthermore, existing sedentary behaviour questionnaires typically focus on either interviewer- [27] or self-administration [21,22]. Interviewer-administered questionnaires are useful for populations with reading difficulties, allowing for clarification, and avoiding questions to be skipped; however, there are drawbacks such aspotential interviewer/social desirability bias and the cost of hiring interviewers [28]. While self-administered methods can facilitate the respondent’s willingness to disclose sensitive information and are less expensive from a staffing viewpoint, there is also a potential for skipping questions, and hence missing data [29,30]. Because the different modes of questionnaire administration could affect the applicability and the reporting of sedentary behaviour [29], it should, therefore, be ensured that the measurement equivalence is determined by comparing the psychometric properties between interviewer- and self-administered questionnaires.

Thus, this study aims to examine: (i) the convergent validity of the ASBQ and the single-item GPAQ against accelerometers in measuring sedentary time, and (ii) the test-retest reliability of the questionnaires with a one-week interval. Both of the aims were assessed among all participants and by the administration mode of the questionnaires (self-administration versus interview-based).

## 2. Materials and Methods

### 2.1. Study Design and Participants

In a cross-sectional study, a convenience sample of an adult population from a large public university and a university hospital of various departments and faculties in Singapore were invited to participate. Participants were invited through printed posters or the university’s mass internal email system. Individuals who wished to participate were followed up by trained research staff in person to administer study materials at their workplace. The inclusion criteria for study participants were Singaporean adults of Chinese, Malay, and Indians ethnic groups, aged 21–65 years, English-literate, and without physical ability or illness that would restrict the performance of normal lifestyle activities. Written informed consent was obtained from each participant. The study was approved by the National University of Singapore Institutional Review Board (NUS-IRB reference number: B-14-021).

According to Prince et al. [31], it was determined that at least 30 participants would be required to achieve adequate statistical power (80%, *α* = 0.05) to detect a moderate correlation (*r* = 0.50) between self-report and objective-based measures.

### 2.2. Procedure

Subjective measures of sedentary behaviour were collected using the domain-specific ASBQ and the GPAQ single-item sitting question; while objective measures were collected using the ActiGraph^TM^ wGT3X-BT accelerometer (LLC, Pensacola, FL, USA). Information on age, gender, ethnicity, educational level, height, and weight were self-reported by participants on a structured questionnaire.

Participants were deskbound population. Before the study began, participants were randomly assigned to two groups to complete either the self- or interviewer-administered questionnaire (Supplementary Materials Figure S1). The randomization sequence was created using computer-generated random assignments (Microsoft Excel, Redmond, WA, USA). For the interviewer-administered questionnaire, interviews were conducted face-to-face by a trained researcher; in the case of the self-administered questionnaire, the participants were asked to complete the questionnaires by hand and return them to the researcher.

On the first day, a researcher explained the objectives of the study and delivered the accelerometers to the participants in person. All of the participants completed the survey at the first appointment. Instructions for wearing the waist-worn accelerometers were provided. They were instructed to wear the accelerometers for seven consecutive days over a 24-h period, while maintaining their usual daily routine. On day seven, a researcher recollected the accelerometers at the participant’s workplace and the participants filled in the retrospective questionnaires. Convergent validity was examined by comparing the retest of the questionnaires against accelerometry-based measures. Test-retest reliability of the questionnaires was determined by comparing the two rounds of measurements.

### 2.3. Subjective Sedentary Behaviour

The ASBQ quantifies the time spent sedentary in a typical week. Adaptation of the existing questionnaires included changing of the wording in the work domain based on questions from the Sit-Q-7d, “In a typical week, how many days do you work or study?”, and “How much time do you usually spend sitting as part of your job (or as part of your studying) while at work (or while at university) or working (or studying) from home?” (The questionnaire of Supplementary Materials). The questionnaire also included a transportation domain (motorized transportation) and a leisure-time sedentary domain (eating meals, watching television, watching videos/surfing the internet/playing electronic games using media devices, such as computer, tablet or mobile phone, and “other activities”) (The questionnaire of Supplementary Materials). The “other activities” domain encompassed sedentary activities including reading, listening to music, socializing, and playing cards. The ASBQ were initially pilot-tested for clarity in 23 participants who were not included in the analysis of the current study.

Time spent in the total and domain-specific sedentary behaviour was calculated for an average day. Average total sitting time (min/day) was calculated based on: (i) a sum of daily domain-specific sedentary time ((workday sitting minutes × 5) + (non-workday day sitting minutes × 2))/7 based on the ASBQ; and (ii) a single-item sitting question, “How much time do you usually spend sitting or reclining on a typical day?” based on the GPAQ. Sleeping during the night was not included in the questionnaire.

### 2.4. Accelerometry-Based Measures

The ActiGraph^TM^ wGT3X-BT (LLC, Pensacola, FL, USA) is a triaxial accelerometer (4.6 × 3.3 × 1.5 cm; 19 g) and has demonstrated over 80% agreement with direct observation for sedentary time [32]. Participants were taught by a trained researcher to put on the ActiGraph for seven consecutive days, and the participants were allowed to remove it while performing water-based activities. The accelerometer was initialized at a sampling rate of 30 Hz. Data were downloaded and integrated into 60-s epochs using the ActiLife software (version 6). The accelerometry vector magnitudes of counts data from all three of the axes were used. Accelerometry-based data was then processed using the Accelerometry Package in R (Version 3.1.3) [33].

Accelerometry wear days were identified by matching against the start/stop dates recorded on participants’ log sheets. To process the 24-h period of accelerometry data, we applied and slightly modified the waist-worn accelerometry-based sleep detection algorithm by Barreira et al. [34] to better reflect adult night-time wear. More detailed descriptions of the data processing and the accelerometer sleep algorithms that were used have been published elsewhere [35]. The remaining wakeful valid accelerometry data were processed using Choi et al. [36] algorithm to define non-wear time: (i) zero-count threshold, (ii) periods of ≥90-min of consecutive zero counts, and (iii) an allowance of ≤2-min interval interruptions (nonzero counts) with a 30-min consecutive zero counts window upstream or downstream of the non-wear period.

A valid day was defined as having an accelerometer wear duration of at least 10 h per day over at least four days, including at least one full weekend day [37]. Sedentary time was defined as all registered accelerometry data (minutes) of <150 counts per minute (CPM) [38]. Sedentary time that were recorded on valid workdays and non-work days were summed and divided by the valid number of days to create an estimate of average daily sedentary time (min/day). Sensitivity analyses were performed using cut points of <100 CPM and <200 CPM as suggested by different validation studies of accelerometer thresholds for sedentary time [38,39].

### 2.5. Statistical Analysis

Descriptive data are presented as means (±standard deviation (SD)) or median (interquartile range (IQR)) for normally and non-normally distributed continuous variables, respectively, and as the number (percentage) for categorical variables.

For each socio-demographic characteristic and variable for subjective and accelerometry-based measures, we performed the Mann-Whitney U test and Chi-square test (or the Fisher’s exact test) to assess the statistical significant differences between the different modes of administration. The Mann-Whitney U test was used for continuous data as most variables (age, questionnaire- and accelerometry-based measures) were not normally distributed. The Chi-square test (or the Fisher’s exact test for categorical variables with cells having an expected frequency of five or less) was used for categorical variables (gender, race, educational level, and work sector).

The convergent validity between the questionnaires and accelerometry-based measures was determined for all of the participants, by self- and interviewer-administered groups. The mean absolute error (MAE) and SD between the questionnaires and accelerometry-based sedentary time were calculated (average of all absolute errors between questionnaires and accelerometry-based sedentary time). The difference in MAE by the modes of questionnaire administration was compared using the Mann-Whitney U test. The Spearman’s rank correlation coefficients test (rho) was used to assess the correlations between the questionnaires and accelerometry-based measures. Spearman’s rho was used due to the non-normal distribution of measures. In addition, the agreement between questionnaire- and accelerometry-based sedentary time was assessed using Bland-Altman plots with the 95% limits of agreement.

The test-retest reliability of each questionnaire was done by calculating the Spearman’s rho and the two-way mixed model (single measure) intraclass correlation coefficient (ICC), with a 95% confidence interval (CI).

To assess whether the correlation coefficients of validity and reliability differed between different modes of administration, a *Z*-test was used [40,41]. To interpret Spearman’s rho, the following benchmarks were used: 0–0.20 poor correlation, 0.21–0.40 fair correlation, 0.41–0.60 moderate/acceptable correlation, 0.61–0.80 good correlation, and 0.81–1.00 strong correlation [42]. ICC values of ≤0.39 are indicative of poor agreement, 0.40–0.59 indicate moderate agreement, 0.60–0.79 indicate good agreement, and ≥0.80 indicate strong agreement [43]. All of the statistical analyses were performed using SPSS version 20.0 (SPSS Inc., Chicago, IL, USA) and Stata version 14.0 software (Stata, College Station, TX, USA) with a significance level set at *p* < 0.05.

## 3. Results

Out of 87 participants that were recruited, a total of 84 participants who completed the ASBQ and answered the GPAQ single-item sitting question were included in the test-retest reliability study (response rate: 96.6%). Of those, 78 participants who met the wear time criteria were included in the validity study (response rate: 89.7%) (Supplementary Materials Figure S1). Participants were predominantly female (69.0%), with an age range of 20 to 65 years (median age: 32.5 years), mostly Chinese (86.9%), with more than three-quarters completed their highest degree from a university (81.0%), mostly full-time employee (86.9%), and mostly being recruited from the public university (58.3%) (Supplementary Materials Table S1). There was a difference in gender distribution between self- and interviewer-administered groups (Female: 57.5% and 79.5%, respectively).

### 3.1. Descriptive Statistics of Subjective Sedentary Behaviour

Average minutes per day being spent in sedentary behaviours, as assessed by the ASBQ and the GPAQ are presented in Supplementary Materials Figure S2. The sum of ASBQ domain-specific sedentary time was higher than the GPAQ single-item sitting time for all of the participants. Occupational sitting domain represented the highest sedentary time as compared to other domains. There was no significant difference in sedentary time between the different modes of questionnaire administration.

### 3.2. Descriptive Statistics of Accelerometry-Derived Data

A detailed account of accelerometry-derived estimates is presented in Table 1. A total of 78 participants had valid accelerometer data (median 6.6 days/week [IQR 6.0–7.0]), with a mean wear time of 15.2 h/day (SD: 1.4). Overall, the participants spent about 73.1% (11.1 h [SD: 1.2]) of their time in sedentary activities. Accelerometry-derived sedentary time was lower among the participants who had completed the self-administered questionnaire as compared to those who completed the interviewer-administered questionnaire. In the sensitivity analyses, higher CPM thresholds constituted to higher sedentary time (Supplementary Materials Table S2). An incremental increase in the relative sedentary time was observed as the CPM thresholds increased. However, as the change in total sedentary time was not substantial, the results were only presented for the cut point of 150 CPM as the main findings.

### 3.3. Convergent Validity of the GPAQ and the ASBQ against Accelerometers

Among all of the participants, only the GPAQ single-item sitting (rho: 0.28) was significantly correlated with accelerometry-based measures. Based on self-administration, a moderate correlation in the GPAQ single-item sitting (rho: 0.46), a fair correlation in the ASBQ eating domain (rho: 0.34), and a correlation of borderline significance for occupational sitting (rho: 0.32) were reported. No significant correlation was observed using interviewer-administered questionnaires. A significantly higher correlation in the validity was reported for the eating domain based on self-administered questionnaire when compared to the interviewer-administered questionnaire (rho: 0.34 vs. −0.17).

Analyses on MAE and validity correlations between the questionnaires and accelerometry-measures are presented in Table 1. The MAE between accelerometry-derived sedentary behaviour and the sum of ASBQ total sedentary time were lower than that reported by the GPAQ sedentary time in all of the participants and across different modes of administration. Within modes of administration, a slightly smaller MAE value was observed for the GPAQ sedentary time using the self-administered questionnaire as compared to the interviewer-administered questionnaire. Conversely, the MAE value was larger when using the self-administered ASBQ as compared to the interviewer-administered ASBQ.

**Figure 1:**
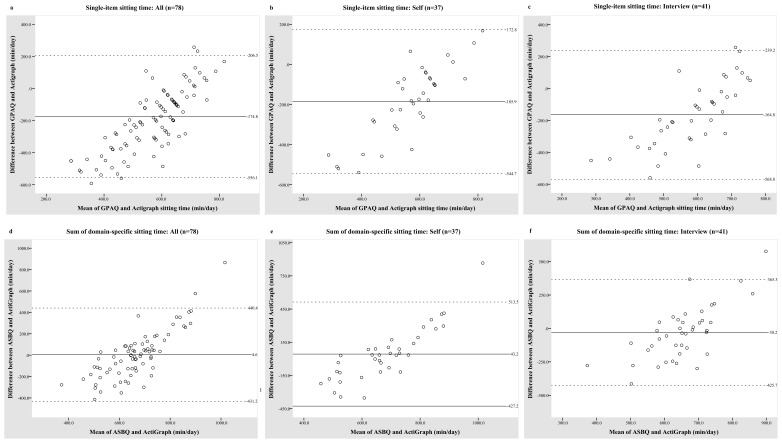
Bland-Altman plots of minutes of sedentary time per day between the questionnaires and ActiGraph GT3X accelerometer. (**a**–**c**) The Global Physical Activity Questionnaire (GPAQ) vs. accelerometry-based measures in all participants, self- and interviewer-administered groups, respectively; (**d**–**f**) the Adult Sedentary Behaviour Questionnaire (ASBQ) vs. accelerometry-based measures in all participants, self- and interviewer-administered groups, respectively.

From the Bland-Altman analysis, sedentary time tended to be underestimated by the GPAQ with respect to the accelerometers (Figure 1). A smaller mean difference was found between the ASBQ and accelerometry as compared to the GPAQ; nonetheless, both the GPAQ and the ASBQ showed large limits of agreement across all of the participants and by different modes of administration. Upward trends that were observed in the Bland-Altman plots indicated that the difference between measurements increases with increasing sedentary time.

### 3.4. Test-Retest Reliability of the Questionnaires

The one-week test-retest reliability correlation coefficients of the GPAQ and the ASBQ are presented in Table 2. There were good correlations (rho: 0.68–0.79) and agreement (ICC: 0.68–0.78) for the GPAQ single-item sitting question across all of the participants and different modes of questionnaire administration. For the sum of ASBQ domain-specific items, there were moderate-to-good correlations (rho: 0.53–0.64) and good agreement (ICC: 0.66–0.74). No significant difference was found in the correlation coefficients between the GPAQ and the ASBQ domain-specific items across different modes of administration.

As for individual items of the ASBQ, a strong correlation (rho: 0.82) and a good agreement (ICC: 0.78) for occupational sitting were shown among all of the participants. The weakest reliability was found for “other activities” among all of the participants, reporting a moderate correlation (rho: 0.41) and a poor agreement (ICC: 0.38). Significant differences across some domains were found between the modes of administration, such as occupational sitting (ICC: 0.70 vs. 0.89 for self- and interviewer-administration, respectively), television viewing (rho: 0.85 vs. 0.55 for self- and interviewer-administration, respectively), and “other activities” (rho: 0.51 vs. 0.37 for self- and interviewer-administration, respectively).

## 4. Discussion

This study evaluated the validity and reliability of the domain-specific ASBQ and the GPAQ single-item sitting question in an Asian population. Only the GPAQ showed a significant correlation with the accelerometry-based sedentary time, especially when using the self-administered mode of the questionnaire. However, both GPAQ and ASBQ showed high MAE in relation to the accelerometer. Although the ASBQ provided a lower MAE when compared to the GPAQ, there was no significant difference in the values between questionnaires and by modes of administration. Also, the Bland-Altman plots demonstrated wide limits of agreement between questionnaire- and accelerometry-based sedentary time using either the GPAQ or the ASBQ. Both of the questionnaires demonstrated moderate-to-good test-retest reliability in assessing the total sedentary time using either the self- or interviewer-administrated version.

The observed significant correlation between the GPAQ and accelerometry, but not the ASBQ indicates that the GPAQ single-item sitting screening question might be useful for ranking participants in terms of their sedentary behaviour levels. A systematic review by Helmerhorst et al. [44] reported that a median Spearman’s rho of 0.23 was typically found between self-report and accelerometry-derived sedentary time. Our findings were similar to most sedentary behaviour questionnaires, which typically reported validity coefficients of between 0.30 and 0.39 when compared with accelerometry [22,45,46,47].

In contrast to our findings of no significant correlation when comparing the sum of ASBQ domain-specific sedentary behaviour against accelerometry, Clemes et al. [48] reported a significant correlation between the total sedentary time that was estimated from domain-specific questionnaire (i.e., at work, time spent sitting while traveling, watching television, using a computer at home, and during leisure-time) and the accelerometry-based sedentary time. Another study by Visser et al. [47] also showed that the total self-reported sedentary time that was derived from the sum of 10 sedentary activities domains was significantly correlated with accelerometry sedentary estimates in older adults (rho: 0.35). In our study, the exploration of domain-specific sedentary time based on the ASBQ and accelerometry sedentary time found significant correlations only for the eating domain based on self-administration. The lack of significant correlations in our study probably reflects the fact that sedentary behaviour patterns across different settings are variable or irregular in nature; therefore, it was more difficult to recall specific sedentary behaviours. There could be a possibility of overlap in different sedentary behaviours together (i.e., eating while watching television), which could have resulted in a misreporting of sedentary time. Future studies should compare self-reported sedentary behaviour against time stamped objective data for a more precise assessment of sedentary time in different contexts.

Correlation coefficients establish the degree to which two measures are related, but not necessarily depict good agreement [49]. In our study, although a correlation was observed between the GPAQ and the accelerometer, the Bland-Altman plots demonstrated wide limits of agreement, showing that the accuracy of GPAQ is perhaps low. Sedentary time that was estimated by the GPAQ tended to be under-reported when compared to accelerometry-derived data. This finding is consistent with previous studies which have compared self-reported and objective sedentary time [46,47,50,51]. Marshall et al. [50] compared total self-reported sedentary time to day-specific GT1M ActiGraph and reported a very low agreement in women, as shown by the Bland-Altman analysis. In our study, although a small mean difference between the ASBQ and accelerometry was observed in the Bland-Altman plot, the MAE and the limits of agreement between the ASBQ and accelerometry-based sedentary time were substantial, too. In addition, there is evidence of bias in which the differences in the sedentary time between questionnaire- and accelerometry-based measures increased with higher amounts of sedentary time. This might reflect the inability of the participants to recall sedentary time well, or a result of participants rounding up of sedentary time using questionnaires [52]. These observations suggest that both ASBQ and GPAQ are probably unable to assess sedentary time on an individual basis accurately, and may not be sufficiently sensitive to detect changes in the sedentary behaviour outcome in intervention studies.

Imprecise measurement of sedentary behaviour levels might attenuate the associations or introduce bias when investigated with health-related outcomes [53,54] and impact studies or national surveillance monitoring of the population-based sedentary time [54]. There is also a need for considering factors that are likely to cause bias when examining subjectively measured sedentary behaviour (e.g., age, gender, musculoskeletal disorders, body weight). In addition, Gupta et al. [51] have employed a statistical predictive model that could help to explain the variance in accelerometry-based sedentary time, and could, therefore, be applied in future studies.

Overall, the questionnaires showed good reliability, which was consistent with previous studies that examined the test-retest of sitting questionnaires of 1 month apart [21], 3.3 weeks apart [22], and 3.4 weeks apart [22]; while our study showed a lower agreement than a reliability study with a two-week interval [20]. These different results might be attributable to the nature of the populations. Higher reliability correlations were observed for sedentary behaviours that vary less from day-to-day and tend to be done on a regular and prolonged basis, such as occupational sitting [21]. The lower values of Spearman’s rho and ICC for reliability in some domain-specific sedentary behaviours (e.g., leisure-time and “other activities” domains) might be explained by the true differences in behaviours. Although the questionnaires recalled a habitual week, the questionnaires were administered at different periods, and the potential recall bias is an inherent issue with subjective methods. Other plausible explanations could also be that a bank holiday was included in the second week, weather variations (e.g., rainy days), or that the respondents were more aware of the questions that were asked upon the first round of questionnaire administration [55].

### Strengths and Limitations

Strengths of our study include the high compliant accelerometer wear of a median of 6.6 days on average, the testing of these questionnaires across different modes of administration, and the analysis of vector magnitude data captured by tri-axial accelerometry, which allows for the detection of accelerations across three axes.

The findings of this study had limited generalizability, as our participants were more representative of those who had higher levels of education and had a full-time job. The triaxial ActiGraph mounted on the waist may have limitations to be used as a reference method to detect sedentary activities (i.e., inability to distinguish between standing still and sitting down). In future investigations, it might be possible to use different objective tools (e.g., the activPAL) that distinguish these movements more accurately. Also, different accelerometry data processing methods will affect the degree of discrepancy between self-reported and accelerometry sedentary time. We applied sensitivity analysis by using strictly at least seven days per week of wear criterion to our data; however, the sample size was reduced substantially to be deemed underrepresented. We have also applied different cut points for determining accelerometry-based sedentary time (i.e., 100 and 200 CPM), reporting similar findings. Furthermore, as daily activities are compositional by nature, future studies should perform compositional data analysis to explore the co-dependent behaviours of sleep, sedentary behaviour, and physical activity [56]. Another limitation is that the accelerometry-derived sedentary estimates might be biased toward underestimation by the application of a 10-h wear time criteria [57]. However, this is unlikely because most of the participants in this study recorded longer accelerometry valid wear time. Further improvement in data quality could be achieved by applying stringent criteria on accelerometry daily wear time in future studies (e.g., ≥14–16 h/day) [58]. Also, with a short interval of one week for the test-retest reliability study, there was a potential that participants recalled and answered the questionnaire based on their past responses but not their actual behaviours.

## 5. Conclusions

In summary, only the GPAQ showed acceptable validity with the accelerometry-based sedentary time, especially when using the self-administered version. However, the potential over- and under-estimation of sedentary time by the GPAQ needs to be taken into account. The ASBQ showed poor convergent validity in assessing the total sedentary time when compared to the accelerometer. Moderate-to-good reliability was found for both the GPAQ and the ASBQ, and the reliability between self- and interviewer-administered modes of questionnaires were found to be comparable. Accurate and valid measures are needed to monitor the population-based sedentary behaviour levels and to determine the associations with health-related outcomes. We suggest that self-reported sedentary behaviour data should be interpreted with caution due to the variability in sedentary activities being performed in general. Future studies should incorporate objective (accelerometry) measures to more accurately assess adults’ sedentary behaviour.

## Figures and Tables

**Table 1:** Accelerometry-derived sedentary time estimates using 150 counts per minute (CPM) and convergent validity of the Global Physical Activity Questionnaire (GPAQ) and the Adult Sedentary Behaviour Questionnaire (ASBQ).

Accelerometry Estimates	All (*n* = 78)	Self (*n* = 37)	Interview (*n* = 41)
Valid wearing day/week (median (IQR))	6.6 (6.0–7.0)	7.0 (6.0–7.0)	6.0 (5.5–7.0)
Valid wear time, min/day (mean ± SD)	910.6 ± 82.2	915.6 ± 84.6	906.1 ± 80.8
Total sedentary time, min/day (mean ± SD)	663.8 ± 70.4	658.6 ± 65.8	668.5 ± 74.8
Relative sedentary time (%) (mean ± SD) ^a^	73.1 ± 6.3	72.1 ± 5.5	73.9 ± 6.8
Spearman’s rho (95% CI)			
GPAQ single-item	0.28 (0.11–0.47) *	0.46 (0.18–0.68) *	0.12 (−0.11–0.33)
ASBQ sum of domain-specific	0.10 (−0.12–0.32)	0.31 (−0.02–0.58)	−0.07 (−0.37–0.24)
Occupational sitting	0.18 (−0.05–0.38)	0.32 (0.03–0.59)	0.11 (−0.20–0.41)
Transportation	0.11 (−0.12–0.32)	0.12 (−0.22–0.42)	0.10 (−0.22–0.39)
Eating	0.07 (−0.16–0.29)	0.34 ^b^ (0.70–0.58) *	−0.17 ^b^ (−0.45–0.15)
Television viewing	0.05 (−0.18–0.26)	0.08 (−0.25–0.40)	0.02 (−0.29–0.32)
Leisure-time computer use	−0.10 (−0.32–0.12)	0.09 (−0.24–0.40)	−0.28 (−0.54–0.03)
Other leisure-time activities	−0.05 (−0.27–0.18)	0.08 (−0.24–0.40)	−0.14 (−0.43–0.17)
MAE ± SD, min/day ^c^			
GPAQ single-item	213.2 ± 150.9	207.5 ± 157.4	218.3 ± 146.5
ASBQ sum of domain-specific	164.1 ± 148.9	174.6 ± 167.8	154.7 ± 130.9

* Statistically significant at *p* < 0.05; ^a^ Total sedentary time divided by total wear time; ^b^ Average of all absolute errors between questionnaire and accelerometry-based sedentary time; ^c^ Significant difference in the correlation coefficients between self- and interviewer-administered groups, *p* < 0.05; Abbreviations: CI, confidence interval; CPM, counts per minute; IQR, interquartile range; MAE, Mean absolute error; SD, standard deviation.

**Table 2:** Test-retest reliability of the Global Physical Activity Questionnaire (GPAQ) and the Adult Sedentary Behaviour Questionnaire (ASBQ) across all of the participants, self- and interviewer-administered groups.

Sedentary Behaviour	All (*n* = 84)	Self (*n* = 40)	Interview (*n* = 44)	All (*n* = 84)	Self (*n* = 40)	Interview (*n* = 44)
	Spearman, rho (95% CI)			ICC (95% CI)		
GPAQ single-item	0.74 (0.62–0.82)	0.68 (0.47–0.82)	0.79 (0.64–0.88)	0.73 (0.61–0.82)	0.68 (0.47–0.82)	0.78 (0.64–0.88)
ASBQ sum of domain-specific	0.61 (0.46–0.73)	0.64 (0.41–0.79)	0.53 (0.28–0.72)	0.72 (0.57–0.82)	0.74 (0.51–0.86)	0.66 (0.37–0.81)
Occupational sitting	0.82 (0.74–0.88)	0.83 (0.70–0.91)	0.79 (0.64–0.88)	0.78 (0.66–0.86)	0.70 ^a^ (0.43–0.84)	0.89 ^a^ (0.80–0.94)
Transportation	0.71 (0.58–0.80)	0.68 (0.47–0.82)	0.71 (0.53–0.83)	0.68 (0.51–0.79)	0.59 (0.22–0.78)	0.78 (0.59–0.88)
Eating	0.59 (0.43–0.71)	0.56 (0.30–0.74)	0.58 (0.34–0.75)	0.73 (0.58–0.82)	0.73 (0.48–0.86)	0.71 (0.47–0.84)
Television viewing	0.78 (0.68–0.85)	0.85 ^a^ (0.73–0.92)	0.55 ^a^ (0.30–0.73)	0.82 (0.73–0.88)	0.85 (0.73–0.92)	0.81 (0.67–0.89)
Leisure-time computer use	0.67 (0.53–0.77)	0.66 (0.45–0.81)	0.67 (0.46–0.80)	0.59 (0.43–0.71)	0.57 (0.32–0.75)	0.62 (0.40–0.78)
Other leisure-time activities	0.41 (0.23–0.58)	0.51 ^a^ (0.24–0.71)	0.37 ^a^ (0.08–0.60)	0.38 (0.18–0.55)	0.33 (0.04–0.57)	0.42 (0.13–0.64)

^a^ Significant difference in the correlation coefficients between self- and interviewer-administered groups, *p* < 0.05. Abbreviations: CI, confidence interval.

## Supplementary Materials

The following are available online at http://www.mdpi.com/1660-4601/15/4/739/s1, Figure S1. Participants flow diagram; Figure S2. Sitting time assessed from the GPAQ single-item sitting question, the sum of domain-specific sitting items from the ASBQ and individual domains from the ASBQ (occupational, transport, eating, television viewing, leisure-time computer use and other sedentary activities); Table S1. Socio-demographic characteristics of study population; Table S2. Accelerometry-derived sedentary time estimates using 100 and 200 CPM.
