# Supplementary material for: Domain-Specific Adult Sedentary Behaviour Questionnaire (ASBQ) and the GPAQ Single-Item Question: A Reliability and Validity Study in an Asian Population
Source: Int J Environ Res Public Health. 2018 Apr 12;15(4):739. doi: 10.3390/ijerph15040739 (PMC5923781; doi:10.3390/ijerph15040739)
Supplement: Supplementary file 1 [file ijerph-15-00739-s001.pdf]

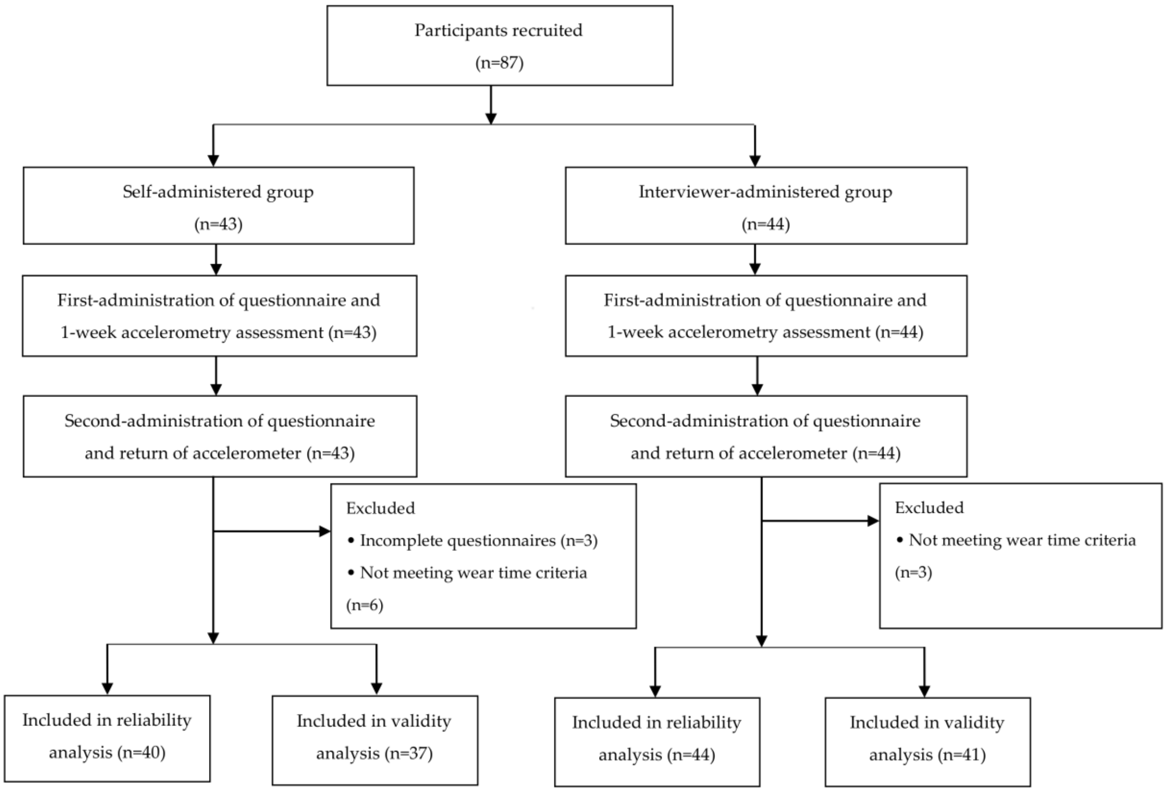

Figure S1. Participants flow diagram.

Sedentary Behaviour Assessment.

**Global Physical Activity Questionnaire (GPAQ)**<br>**Single-item sitting question**		
The following question is about sitting or reclining at work, at home, getting to and from places, or with friends, including time spent [sitting at a desk, sitting with friends, travelling in car, bus, train, reading, playing cards or watching television], but **do not include time spent sleeping during the night**.		
Q1	How much time do you usually spend sitting or reclining on a typical day?	Hours : minutes<br>└─┴─┘ : └─┴─┘
**Adult Sedentary Behaviour Questionnaire (ASBQ)**		
**Sitting as part of work/studying**		
Q2	In a typical week, on how many days do you work or study?	____ days a week
Q3	How much time do you usually spend sitting as part of your job (or as part of your studying) while at work (or while at university) or working (or studying) from home?	Hours : minutes<br>└─┴─┘ : └─┴─┘

We would like to know how much time you spend sitting or reclining during **activities not related to work**. Please indicate the amount of time spent sitting or reclining during these activities on working and non-working days.

		Working day		Non-working day	
**Transportation**		Days /week	Hours : minutes (per day)	Days /week	Hours : minutes (per day)
Q4	Sitting during motorized transport to go to and from places	___	___ : ___	___	___ : ___

**Leisure Time**					
Q5	a. Eating meals (including breakfast, lunch and dinner)	___	___ : ___	___	___ : ___
Q5	b. Television viewing (i.e., Watching TV, DVD)	___	___ : ___	___	___ : ___
Q5	c. Watching video (i.e., YouTube, online video), Internet surfing, social media, electronic games on any media device e.g., on <u>computer</u>, <u>tablet</u> or <u>mobile phone</u>	___	___ : ___	___	___ : ___
Q5	d. Other leisure time activities such as sitting and reading or listening to music, playing cards, using telephones, socializing with friends or family (please do NOT include the time you mentioned in the previous question)	___	___ : ___	___	___ : ___

This is the end of the questionnaire.

Thank you!

Table S1. Socio-demographic characteristics of study population.

Characteristics		All (*n* = 84)	Self (*n* = 40)	Interview (*n* = 44)	*P*-value*
Gender, n (%)					0.03
	Male	26 (31.0)	17 (42.5)	9 (20.5)	
	Female	58 (69.0)	23 (57.5)	35 (79.5)	
Age, median (IQR)		32.5 (27.0–46.8)	33.0 (26.0–51.0)	31.5 (27.3–45.8)	0.92
Race, n (%)					
	Chinese	73 (86.9)	34 (85.0)	39 (88.6)	0.60

	Malay	3 (3.6)	1 (2.5)	2 (4.5)	
	Indian	8 (9.5)	5 (12.5)	3 (6.9)	
Educational level, n (%)					0.11
	Secondary	3 (3.5)	1 (2.5)	2 (4.6)	
	Technical school/Diploma	13 (15.5)	3 (7.5)	10 (22.7)	
	University	68 (81.0)	36 (90.0)	32 (72.7)	
					0.24
Occupation, n (%)	Full-time employee	73 (86.9)	34 (85.0)	39 (88.6)	
	Student	11 (13.1)	6 (15.0)	5 (11.4)	
Work sector, n (%)					0.10
	University Hospital	35 (41.7)	27 (67.5)	22 (50.0)	
	Public University	49 (58.3)	13 (32.5)	22 (50.0)	

*Significance difference between self- and interviewer-administered versions. IQR, interquartile range; SD, standard deviation.

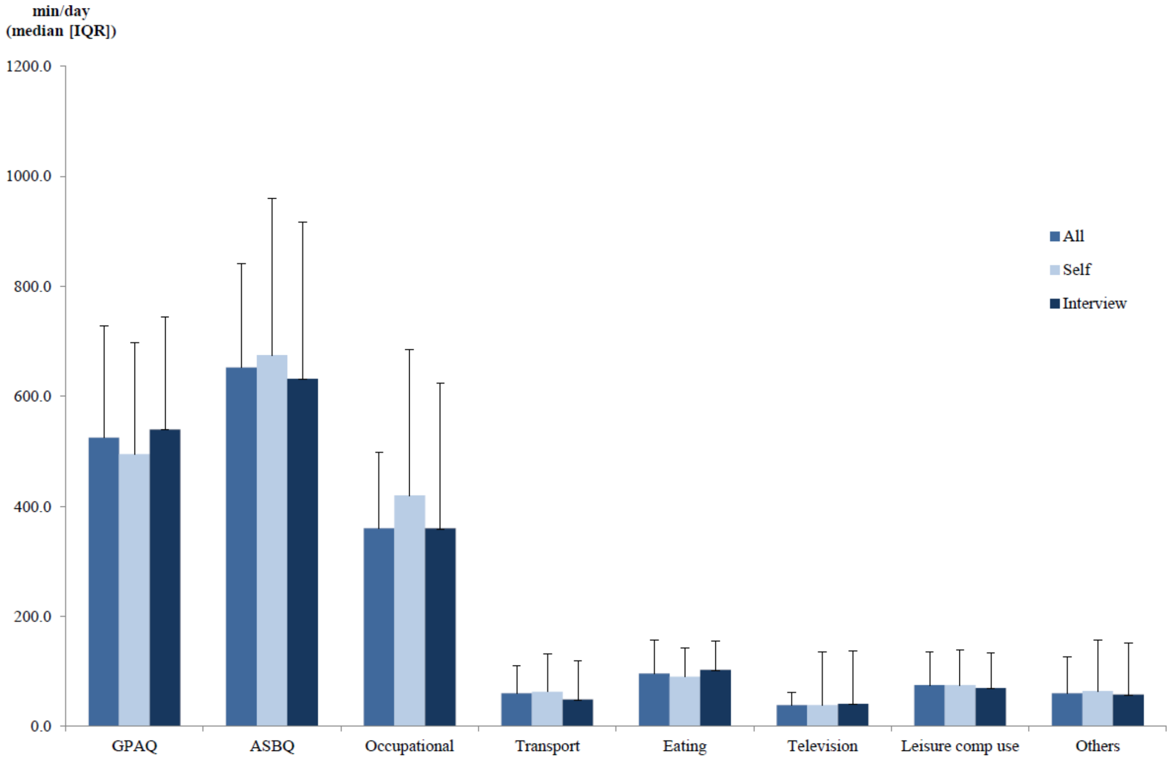

Figure S2. Sitting time assessed from the GPAQ single-item sitting question, the sum of domain-specific sitting items from the ASBQ and individual domains from the ASBQ (occupational, transport, eating, television viewing, leisure-time computer use and other sedentary activities).

Table S2. Accelerometry-derived sedentary time estimates using 100 and 200 CPM.

Accelerometry estimates (100 CPM)	All (n = 78)	Self (n = 37)	Interview (n = 41)
**100 CPM**			
Total sedentary time, min/day (mean ± SD)	629.2 ± 70.4	623.6 ± 66.4	634.2 ± 74.2
Relative sedentary time (%) (mean ± SD)[a]	69.3 ± 6.8	68.3 ± 6.1	70.2 ± 7.4
**200 CPM**			
Total sedentary time, min/day (mean ± SD)	689.6 ± 70.6	684.8 ± 66.0	694.0 ± 75.0
Relative sedentary time (%) (mean ± SD)[a]	75.9 ± 5.8	74.9 ± 5.1	76.7 ± 6.3

[a] Total sedentary time divided by total wear time. CPM, counts per minute; IQR; SD, standard deviation.
